# Investigating the Association Between Anemia and Biochemical Factors in Relation to New Anthropometric Metrics in an Iranian Cohort: A Cross‐Sectional Study

**DOI:** 10.1002/hsr2.72867

**Published:** 2026-07-30

**Authors:** Maryam Kohsari, Zohreh Rahimi, Mehdi Moradinazar

**Affiliations:** ^1^ Social Development and Health Promotion Research Center, Health Institute Kermanshah University of Medical Sciences Kermanshah Iran; ^2^ Department of Clinical Biochemistry Kermanshah University of Medical Sciences Kermanshah Iran

**Keywords:** anemia, anthropometric indices, biochemical parameters, obesity

## Abstract

**Background and Aims:**

Obesity has become one of the most significant health issues worldwide, and the relationship between obesity and anemia remains controversial. This study aims to assess the relationship between lipid‐based obesity parameters, new anthropometric indices, and anemia in the Kurdish population of Iran.

**Methods:**

9810 participants (48.2% male and 51.8% female) were evaluated in the present study. Study data were collected from the initial phase of the Ravansar non‐communicable disease cohort study (RaNCD). All anthropometric and laboratory factors were measured using standard guidelines and methods.

**Results:**

The prevalence of anemia was 8.95%, with 72% of cases in females and 27% in males. No significant difference was observed in body mass index (BMI) between groups. Anemic individuals exhibited significantly higher levels of waist circumference (WC), hip circumference (HC), weight‐adjusted weight (WWI), body adiposity index (BAI), and a body shape index (ABSI) compared to non‐anemic individuals. In contrast to BMI, these parameters showed a negative association with RBC count, HCT, and HB. ABSI presented the highest odds for anemia (OR = 2.26; 95% CI: 1.68–3.03), followed by lipid accumulation product (LAP) (OR = 1.6 in males and 1.55 in females), BAI (OR = 1.39), and HC (OR = 1.34). BMI was conversely associated with anemia (OR = 0.71; 95% CI: 0.51–0.93). Quadratic fit plots by gender revealed a positive correlation between BMI and HGB levels in males with BMI < 30. Additionally, WC and HC in females and WWI and ABSI in males were negatively correlated with Hb levels.

**Conclusion:**

Current results suggest that the shape of obesity and local fat accumulation may have a more significant association with anemia. Therefore, anthropometric indices reflecting localized obesity could be more practical parameters for anemia assessment.

## 1 Introdhuction

Anemia is defined as a reduction in red blood cells (RBC) and lower hematocrit (HCT) and hemoglobin (HGB) levels than the normal range. Individuals with anemia experience hypoxia in body tissues and may suffer complications such as dizziness, headaches, rapid heart rate, hypertension, and neurological disorders [1, 2, 3].

The etiology of anemia is multifactorial [1]. Obesity, particularly body mass index (BMI), has been studied in various populations to evaluate its association with anemia. However, the results obtained have been contradictory. In a Chinese population, Qin et al. reported that overweight and obese individuals had a lower risk of anemia [4]. In contrast, some studies have reported opposite findings in this regard [5]. Additionally, Aboromia et al. found that elevated cholesterol levels in Egyptian patients with dyslipidemia were associated with iron deficiency anemia [6]. Conversely, Ausk and Ioannou reported no significant effect of increased BMI on hemoglobin levels compared with normal BMI [7].

Obesity has become one of the most significant health problems in modern societies and has been progressively increasing worldwide. It is known to contribute to metabolic disorders such as cardiovascular disease, diabetes, and non‐alcoholic fatty liver disease (NAFLD) [8]. It is also associated with low‐grade inflammation, which may increase hepcidin synthesis and lead to anemia [9]. Moreover, previous findings have indicated that obesity and elevated BMI, through the development of NAFLD, are associated with iron deficiency anemia [10].

This study seeks to examine the relationship between anemia and novel biochemical and anthropometric indices in the Kurdish population of western Iran, in light of conflicting findings in Iran, where studies indicate no association between obesity and anemia in the northern region [11] but a significant relationship between increased BMI and anemia in the central region [12], yet a significant correlation between elevated BMI and anemia in the central region.

## 2 Materials and Methods

This study employed data from the initial phase of the Ravansar Non‐Communicable Disease (RaNCD) cohort study, gathered between 2014 and 2017. The criteria and extensive details were thoroughly documented in the previously published publications [13, 14], and at http://Persiancohort.com.

Blood indices and biochemical parameter measurements:

The whole blood and serum were collected after at least 8 h of fasting. A Sysmex cell counter (Sysmex, USA) was used for the CBC differentiation test. The biochemical parameters, including fasting blood sugar (FBS); lipid profile (total cholesterol (TC), triglyceride (TG), low‐density lipoprotein (LDL‐C), and high‐density lipoprotein (HDL)); liver function test (alanine & aspartate aminotransferases (ALT, AST), alkaline phosphatase (ALP), and gamma glutamyl transferase (GGT)); and kidney function tests (urea, creatinine (Cr), and glomerular filtration (GFR)), were measured by the Mindray BS‐380 auto‐analyzer (Mindray, China).

Anemia definition: Anemia was defined based on the WHO guidelines, which consider hemoglobin (Hb) levels < 13 g/dL for males and < 12 g/dL for females [15, 16].

## 3 Demographic Features

Smoking status was categorized according to the National Health Insurance Scheme (NHIS) [17]. Metabolic Equivalent Tasks (METs), by self‐report of their sport, work, and leisure‐related activities for 24 h per weekday, were calculated [18]. Information on alcohol consumption was obtained from individuals’ questions.Measuring blood pressure (systolic (SBP) and diastolic (DBP)) was done by the German Duplex‐Rister, and finally, the mean of two measurements was obtained. The healthy eating index (HEI) was calculated based on the last revised HEI available from the Department of Agriculture, Center for Nutrition Policy and Promotion [19]. The dietary inflammation index (DII), derived from a food frequency questionnaire (FFQ), considered various food parameters such as calories, saturated fat, omega‐3, flavonoids, etc [20]. For measuring anthropometric indices such as weight and height, waist circumference (WC), and hip circumference (HC), a bio‐impedance Analyzer BIA (InBody 770; BIOSPACE, KOREA) and a stadiometer with 0.1 cm accuracy were utilized.

Anthropometric and parameters, including biochemical and anthropometric indices formulas [21]:

- Body mass index(BMI): weight (kg)/height (m^2)^
- Weight‐adjusted‐waist index (WWI): WC (cm)/√weight(kg)
- A body shape index (ABSI): WC/BMI^2/3^ × heigh^1/2^
- Body adiposity index (BAI): [hip circumference (cm) ÷ height (m) 1.5]−18
- Lipid accumulation product (LAP):

Male LAP=[waist(cm)−65]×TG concentration(mmol/l)

Female LAP=[waist(cm)−58]×TG concentration(mmol/l)

- Cardiometabolic index (CMI): TG/HDL‐C × (Waist‐to‐height)
- Atherogenic index of plasma (AIP): Log (TG/HDL‐C)
- The Visceral Adiposity Index (VAI) [22]:

Male VAI=(WC(cm)/(39,68+(1.88∗BMI)∗(TG/1.03)∗(1.31/HDL)

Female VAI=(WC(cm)/(36,58+(BMI∗1.89)∗(TG/0.81)∗(1.52/HDL)

## 4 Criteria for Exclusion

Participants with underlying physiological conditions (pregnancy) and pathological disorders (including cancer, Hepatitis B and C, chronic renal dysfunction, and inflammatory diseases such as rheumatoid arthritis) that affect RBC indices were excluded from the study. Following the application of the exclusion criteria, a total of 9810 participants were enrolled.

## 5 Statistical Analysis

Quantitative data were presented as mean ± SD and analyzed using a two‐tailed T‐test. Qualitative variables were presented as frequencies and percentages, and the chi‐square test was used for analysis. A multivariable linear regression was employed to assess the relationship between RBC indices and various anthropometric parameters. The linear regression model was adjusted for gender, age, menopausal status, blood pressure (SBP and DBP), ALT, GGT, urea, Cr, HEI, and DII. Additionally, a multivariable logistic regression model, including RDW‐CV and the confounder variables of the linear regression model, was used to assess the link between lipid‐based obesity and anthropometric variables and the odds ratio of anemia according to the quartiles of both anthropometric and lipid‐based obesity indices. The best model was chosen based on model fit indices (AIC and BIC). RDW‐CV was not included in the linear regression to prevent overadjustment. Quadratic fit plots were used to compare the effect of lipid‐based obesity indices and anthropometric parameters on Hb based on gender. A *p* value < 0.05 was considered statistically significant. Data analysis was conducted using Stata software (version 14.2, Stata Corp, College Station, TX, USA).

## 6 Ethical Consideration

This study is in accordance with the principles and guidelines of the Helsinki Declaration and got approval from the ethics committees of Kermanshah University of Medical Sciences (KUMS), Kermanshah, Iran (KUMS.REC.1394.315). All the individuals became aware of the study approach and signed the written consent before enrolling in the investigation.

## 7 Results

Table 1 presents the demographic and laboratory information for the participants. The prevalence of anemia among participants was 8.95% (72.7% female vs. 27.3% male). Among the 638 anemic women, 21.5% were in the postmenopausal period. Most participants reported low to moderate levels of physical activity. The HEI was higher in anemic participants (*p* = 0.001), but red meat consumption and DII showed no difference between the anemic and non‐anemic groups. The non‐anemic group exhibited significantly higher SBP (*p* = 0.01) and DBP (*p* < 0.001). Biochemical analysis showed that anemic individuals had lower levels of FBS, lipid profile, liver function, and kidney function tests compared with the non‐anemic group. However, there was no statistical difference between groups in GFR and HDL‐C. Regarding RBC indices, only RDW‐CV was significantly increased in anemic individuals, whereas the other indices (RBC, HCT, Hb, MCV, MCH, and MCHC) were lower than those in the controls (*p* < 0.001).

**Table 1:** General information and laboratory biomarkers of participants.

Variables		Non‐anemic group *N*(%) = 8932 (91.05)	Anemic group *N*(%) = 878 (8.95)	*p*‐value
Gender	Male	4487 (50.2)	240 (27.3)	< 0.001
	Female	4445 (49.8)	638 (72.7)	
Age groups	35–45	4243 (47.5)	389 (44.3)	0.001
	46–55	2817 (31.5)	331 (37.7)	
	56–65	1872 (21)	158 (18)	
Smoking status	Non‐smoker	3526 (40.90)	354 (41.5)	0.85
	Current‐smoker	1008 (11.7)	106 (12.4)	
	Former‐smoker	761 (8.8)	74 (8.7)	
	Passive‐smoker	3327 (38.9)	318 (37.4)	
Alcohol consumption	Yes	8332 (93.3)	851 (96.9)	< 0.001
	No	600 (6.7)	27(3.1)	
Physical activity(METs)	Low	2653 (24.9)	259 (29.7)	< 0.001
	Moderate	4243 (47.9)	484 (55.6)	
	High	1964 (22.2)	128 (14.7)	
Menopausal status	Yes	32.3	21.5	0.001
	No	67.7	78.6	
HEI		51.5 ± 7.31	52.54 ± 7.46	0.001
DII		−2.32 ± 1.60	−2.40 ± 1.49	0.13
Red meat use (g)		22.7 ± 31.1	21.6 ± 29.0	0.11
SBP (mmHg)		108.51 ± 16.92	106.94 ± 18.09	0.01
DBP (mmHg)		70.08 ± 9.95	68.03 ± 9.87	< 0.001
*Laboratory Biomarkers*				
FBS (g/dl)		97.32 ± 30.83	94.47 ± 22.43	0.01
TC (g/dl)		187.15 ± 37.81	169.53 ± 36.15	< 0.001
TG (g/dl)		139.77 ± 85.45	118.04 ± 70.36	< 0.001
HDL‐C (g/dl)		46.38 ± 11.33	46.74 ± 11.40	0.36
LDL‐D (gr/dl)		103.24 ± 25.35	90.82 ± 23.70	< 0.001
ALT (IU)		25.30 ± 14.84	20.71 ± 12.82	< 0.001
AST(IU)		21.61 ± 8.52	19.77 ± 11.53	< 0.001
GGT(IU)		25.00 ± 20.30	20.41 ± 16.60	< 0.001
ALP(IU)		198.55 ± 62.97	189.92 ± 63.33	0.001
Urea(g/dl)		13.65 ± 4.04	13.22 ± 5.63	0.003
Cr (g/dl)		1.00 ± 0.18	0.93 ± 0.46	0.001
GFR		75.89 ± 11.05	77.09 ± 10.86	0.63
RBC count (10^6^/μL)		4.93 ± 0.54	4.76 ± 0.80	< 0.001
HCT (%)		40.15 ± 3.61	32.87 ± 3.14	< 0.001
HGB (g/dl)		14.46 ± 1.30	11.22 ± 1.07	< 0.001
MCH (pg)		29.44 ± 2.42	24.12 ± 4.14	< 0.001
MCHC (g/dl)		36.03 ± 1.35	34.18 ± 1.83	< 0.001
MCV (fL)		81.68 ± 5.65	70.37 ± 10.22	< 0.001
RDW‐CV (%)		10.91 ± 0.82	12.15 ± 1.51	< 0.001

In Table 2, the anemic group showed significantly lower mean AIP and VAI values in both males and females. In contrast, WC, HC, WWI, BAI, and ABSI parameters were significantly higher in anemic individuals than in non‐anemic participants.

**Table 2:** The lipid‐based obesity and anthropometric indices of participants.

Variables		Non‐anemia	anemia	*p*‐value
**Lipid‐based obesity indices**				
AIP		0.99 ± 0.64	0.82 ± 0.60	< 0.001
CMI		1.99 ± 1.62	1.98 ± 1.41	0.91
VAI	Male	4.57 ± 3.76	3.78 ± 3.06	< 0.001
	Female	6.73 ± 5.54	5.56 ± 4.51	< 0.001
LAP	Male	51.77 ± 37.94	51.51 ± 32.61	0.84
	Female	62.59 ± 43.82	62.45 ± 38.18	0.92
**Anthropometrics indices**				
BMI		27.45 ± 4.62	27.7 ± 4.93	0.06
WC		97.21 ± 10.44	98.51 ± 11.12	0.002
HC		102.48 ± 8.76	103.69 ± 9.51	0.001
WWI		11.43 ± 0.83	11.73 ± 0.81	0.01
ABSI		0.84 ± 0.05	0.85 ± 0.05	0.03
BAI		24.04 ± 4.22	25.33 ± 4.52	< 0.001

Table 3 displays the outcomes of the multivariable regression analysis. Elevated AIP and VAI in both genders correlated with increased levels of RBC count, HCT, and HGB; these correlations were statistically significant as indicated by the confidence intervals and the P for trend, especially for RBC count, HCT, and the relationship between AIP and HGB. The confidence intervals for HGB in VAI were significant just in Q4 for both genders (*β*: 0.14; 95%CI: 0.03_0.25 and *P* for Trend = 0.01), despite the *P* for trend retaining significance. The correlation between CMI and LAP (both genders) was inversely related to RBC indices; elevated CMI and LAP resulted in diminished levels of RBC count, HCT, and HGB. In addition, we observed a significant positive association between CMI and MCH, MCHC, and MCV (all P for trend < 0.05). Regarding RDW‐CV, our results showed a positive trend. All lipid‐based obesity indicators have positively influenced the RDW‐CV level. Most outcomes in Q2 and Q3 exhibited significant confidence intervals, and the P for Trend for all parameters was statistically significant (*P* for Trend = 0.001 or < 0.001).

**Table 3:** Multivariable linear regression analysis of lipid‐based obesity indices and RBC indices.

Variables		RBC indices												
		*RBC count*	*HCT*			*HGB*	*MCH*			*MCHC*	*MCV*			*RDW‐CV*
***Lipid‐based Obesity Indices (β; 95% CI)***														
AIP	Q2*	0.06 (0.01_0.10)	0.20 (0.03_0.44)			0.08 (0.005_0.18)	−0.23 (−0.45_−0.009)			0.02 (−0.08_0.13)	−0.71 (−1.22_0.20)			0.11 (0.03_0.19)
	Q3	0.08 (0.04_0.11)	0.28 (0.04_0.53)			0.08 (0.009_0.18)	−0.30 (−0.53_−0.007)			−0.05 (−0.16_0.06)	−0.76 (−1.29_0.23)			0.18 (0.10_0.26)
	Q4	0.10 (0.06_0.14)	0.49 (0.22_0.77)			0.18 (0.0.07_0.29)	−0.22 (−0.47_0.02)			0.01 (−0.11_0.13)	−0.67 (−1.25_0.08)			0.20 (0.11_0.29)
P for Trend		< 0.001	0.003			0.001	0.06			0.80	0.02			< 0.001
CMI	Q2	−0.07 (−0.10_−0.03)	−0.38 (−0.63_−0.12)			−0.12 (−0.22_−0.02)	0.13 (−0.10_0.37)			0.04 (−0.06_0.16)	0.31 (−0.23_0.87)			−0.13 (−0.21_−0.04)
	Q3	−0.14 (−0.16_−0.07)	−0.44 (−0.70_−0.18)			−0.12 (−0.22_−0.02)	0.3 5 (0.11_0.59)			0.10 (−0.002_0.22)	0.79 (0.23_1.24)			−0.16 (−0.25_−0.08)
	Q4	−0.12 (−0.16_0.08)	−0.41 (−0.67_0.15)			−0.10 (−0.20_−0.01)	0.49 (0.25_0.73)			0.13 (0.01_0.24)	1.13 (0.57_1.70)			0.03 (−0.06_0.10)
P for Trend		< 0.001	0.002			0.05	0.01			0.01	< 0.001			< 0.001
VAI−m	Q2	0.07 (0.04_0.10)	0.19 (−0.04_0.43)			0.04 (−0.05_0.13)	−0.34 (−0.56_−0.12)			−0.07 (−0.18_0.03)	−0.80 (−1.31_−0.28)			0.11 (0.04_0.19)
	Q3	0.07 (0.04_0.10)	0.28 (0.03_0.53)			0.06 (−0.03_0.16)	−0.31 (−0.55_−0.08)			−0.10 (−0.22_0.005)	−0.66 (−1.19_−0.12)			0.16 (0.08_0.24)
	Q4	0.09 (0.05_0.13)	0.46 (0.18_0.74)			0.14 (0.03_0.25)	−0.24 (−0.50_0.01)			−0.05 (−0.17_0.07)	−0.55 (−1.15_0.03)			0.17 (0.07_0.19)
P for Trend		< 0.001	0.008			0.01	0.08			0.34	0.1			0.001
VAI‐f	Q2	0.07 (0.04_0.10)	0.18 (−0.04_0.42)			0.04 (−0.05_0.13)	−0.34 (−0.56_−0.11)			−0.07 (−0.18_0.03)	−0.79 (−1.30_−0.27)			0.11 (0.04_0.19)
	Q3	0.07 (0.04_0.10)	0.29 (0.04_0.51)			0.06 (−0.03_0.16)	−0.31 (−0.54_−0.08)			−0.10 (−0.21_0.007)	−0.64 (−1.18_−0.11)			0.16 (0.08_0.24)
	Q4	0.09 (0.05_0.13)	0.45 (0.17_0.74)			0.14 (0.03_0.25)	−0.24 (−0.50_0.009)			−0.05 (−0.18_0.07)	−0.56 (−1.15_0.03)			0.17 (0.07_0.19)
P for Trend		< 0.001	0.008			0.01	0.08			0.33	0.1			0.001
LAP‐m	Q2	−0.13 (−0.16_−0.09)	−0.70 (−0.96_−0.44)			−0.27 (−0.37_−0.17)	−0.02 (−0.20_0.15)			−0.08 (−0.17_0.07)	0.17 (−0.25_0.56)			−0.34 (−0.43_−0.26)
	Q3	−0.20 (−0.23_−0.16)	−0.90 (−1.16_−0.64)			−0.28 (−0.38_−0.17)	−0.06 (−0.25_0.11)			−0.19 (−0.28_0.10)	0.33 (−0.14_0.75)			−0.26 (−0.34_−0.17)
	Q4	−0.21 (−0.24_−0.17)	−0.76 (−1.03_−0.49)			−0.20 (−0.31_−0.10)	0.17 (−0.01_0.36)			−0.11 (−0.20_0.01)	0.77 (0.30_1.11)			0.02 (−0.13_0.03)
P for Trend		< 0.001	< 0.001			0.003	0.002			< 0.001	0.83			< 0.001
LAP‐f	Q2	−0.11 (−0.14_−0.07)	−0.59 (−0.85_−0.33)			−0.22 (−0.32_−0.11)	−0.01 (−0.19_0.16)			−0.03 (−0.12_0.05)	0.03 (−0.38_0.34)			−0.30 (−0.39_−0.22)
	Q3	−0.18 (−0.22_−0.14)	−0.76 (−1.03_−0.50)			−0.23 (−0.33_−0.12)	−0.09 (−0.27_0.09)			−0.18 (−0.28_−0.09)	0.19 (−0.27_0.62)			−0.26 (−0.35_−0.18)
	Q4	−0.18 (−0.22_−0.15)	−0.66 (−0.93_−0.39)			−0.17 (−0.28_−0.07)	0.20 (0.01_0.39)			−0.07 (−0.15_0.04)	0.78 (0.33_1.22)			0.03 (−0.12_0.04)
P for Trend		< 0.001	< 0.001			0.001	0.01			< 0.001	0.77			< 0.001

* Quartile 1 was considered as the reference.

The regression analysis between anthropometric and RBC indices is presented in Table 4. Overall, an inverse association was observed. Higher HC, WWI, BAI, and ABSI were associated with lower RBC count and HCT. In addition, higher WC, HC, and ABSI were associated with lower HGB, MCH, and MCHC. BMI showed a positive association with RBC count (Q4: 0.08; 95% CI: 0.04–0.12; *P* for trend = 0.001) and HCT (Q4: 0.36; 95% CI: 0.09–0.62; *P* for trend = 0.006) but an inverse association with MCH, MCHC, and MCV. All anthropometric parameters, except for WWI (Q4: −0.14; 95% CI: −0.22 to −0.06; *P* for trend < 0.001), showed a positive trend with significant confidence intervals, particularly in Q3 and Q4. Moreover, WWI, BAI, and ABSI were positively associated with MCV (*P* for trend < 0.001). WWI was the only parameter that showed a significant positive association with MCH (Q4: 0.23; 95% CI: 0.05–0.42; *P* for trend = 0.001).

**Table 4:** Multivariable linear regression analysis of anthropometric indices and RBC indices.

*Variables*		*RBC Indices*						
		*RBC count*	*HCT*	*HGB*	*MCH*	*MCHC*	*MCV*	*RDW‐CV*
***Anthropometric indices (β; 95% CI)***								
BMI	Q2*	0.05 (0.02_0.09)	0.23 (−0.05_0.51)	0.05 (−0.04_0.17)	−0.06 (−0.19_0.06)	−0.12 (−0.19_−0.12)	−0.11 (−0.51_0.30)	0.05 (−0.01_0.14)
	Q3	0.06 (0.02_0.10)	0.32 (0.04_0.59)	0.03 (−0.07_0.13)	−0.21 (−0.34_−0.09)	−0.25 (−0.34_−0.16)	−0.30 (−0.86_0.001)	0.15 (0.12_0.24)
	Q4	0.08 (0.04_0.12)	0.36 (0.09_0.62)	0.01 (−0.08_0.12)	−0.28 (−0.40_−0.16)	−0.29 (−0.37_−0.20)	−0.39 (−0.88_−0.09)	0.21 (0.18_0.30)
P for Trend		0.001	0.006	0.9	< 0.001	< 0.001	0.01	< 0.001
WC	Q2	0.03 (−0.02_0.07)	0.10 (−0.14_0.39)	−0.01 (−0.12_0.08)	−0.24 (−0.49_0.001)	−0.16 (−0.28_−0.04)	−0.31 (−0.88_0.26)	0.04 (−0.03_0.12)
	Q3	−0.005 (−0.04_0.03)	−0.08 (−0.3_0.12)	−0.10 (−0.20_−0.06)	−0.01 (−0.26_−0.22)	−0.18 (−0.30_−0.07)	0.41 (−0.14_0.96)	0.10 (0.01_0.18)
	Q4	−0.05 (−0.07_−0.004)	−0.11 (−0.34_0.17)	−0.25 (−0.34_−0.14)	−0.50 (−0.74_−0.26)	−0.50 (−0.62_−0.39)	−0.20 (−0.75_0.33)	0.20 (0.12_0.29)
P for Trend		0.08	0.09	< 0.001	0.008	< 0.001	0.4	< 0.001
HC	Q2	−0.03 (−0.07_0.003)	−0.16 (−0.43_0.09)	−0.08 (−0.19_0.01)	−0.02 (−0.22_0.26)	−0.06 (−0.18_0.05)	0.21 (−0.35_0.78)	−0.09 (−0.11_0.07)
	Q3	−0.03 (−0.06_0.09)	−0.16 (−0.42_0.09)	−0.15 (−0.25_−0.05)	−0.16 (−.40_0.08)	−0.22 (−0.33_−0.10)	0.09 (−0.47_0.65)	0.03 (0.04_0.12)
	Q4	−0.05 (−0.08_−0. 10)	−0.26 (−0.51_0.01)	−0.24 (−0.34_−0.14)	−0.24 (−0.47_−0.01)	−0.37 (−0.48_−0.26)	0.21 (−0.31_0.75)	0.14 (0.06_0.222)
P for Trend		0.01	0.04	< 0.001	0.01	< 0.001	0.5	0.002
WWI	Q2	−0.05 (−0.09_−0.02)	−0.33 (−0.59_−0.07)	−0.05 (−0.15_−0.04)	0.12 (0.02_0.29)	−019 (−0.26_−0.08)	1.2 (0.65_1.77)	−0.05 (−0.10_0.02)
	Q3	−0.06 (−0.10_−0.02)	−0.31 (−0.56_−0.05)	−0.09 (−0.19_0.01)	0.21 (0.05_0.43)	−0.24 (−0.30_−0.14)	1.5 (1.00_2.13)	−0.13 (−0.17_−0.09)
	Q4	−0.14 (−0.16_−0.09)	−0.42 (−0.68_−0.16)	−0.11 (−0.21_−0.09)	0.23 (0.05_0.42)	−0.36 (−0.44_−0.27)	2.0 (1.47_2.63)	−0.14 (−0.22_−0.06)
P for Trend		< 0.001	0.003	0.1	0.001	< 0.001	< 0.001	< 0.001
BAI	Q2	−0.10 (−0.13_−0.06)	−0.22 (−0.48_0.03)	−0.02 (−0.12_0.08)	0.12 (−0.01_0.28)	−0.14 (−0.17_−0.01)	0.21 (−0.33_0.76)	−0.47 (−0.56_−0.38)
	Q3	−0.14 (−0.17_−0.10)	−0.38 (−0.65_−0.12)	−0.06 (−0.17_0.03)	0.05 (−0.11_0.29)	−0.31 (−0.39_−0.19)	0.41 (−0.13_0.97)	−0.21 (−0.29_−0.12)
	Q4	−0.18 (−0.22_−0.14)	−0.52 (−0.79_−0.25)	−0.05 (−0.15_0.04)	−0.04 (−0.27−0.19)	−0.44 (−0.56_−0.37)	1.22 (0.69_1.81)	−0.11 (0.20_−0.06)
P for Trend		< 0.001	0.001	0.33	0.03	< 0.001	< 0.001	< 0.001
ABSI	Q2	−0.08 (−0.11_−0.04)	−0.38 (−0.65_−0.11)	−0.21 (−0.31_−0.10)	0.30 (0.04_0.55)	−0.17 (−0.29_−0.05)	0.55 (−0.03_1.13)	−0.32 (−0.40_0.22)
	Q3	−0.16 (−0.20_−0.13)	−0.86 (−1.12_−0.59)	−0.39 (−0.49_−0.28)	0.17 (0.07_0.42)	−0.21 (−0.33_−0.09)	1.00 (0.42_1.57)	−0.23 (−0.32_−0.15)
	Q4	−0.22 (−0.24_−0.16)	−0.96 (−1.23_−0.69)	−0.43 (−0.53_−0.31)	0.04 (−0.2_0.03)	−0.18 (−0.30_−0.06)	1.30 (0.73_1.80)	−0.13 (−0.23_−0.16)
P for Trend		< 0.001	< 0.001	< 0.001	0.01	0.003	< 0.001	< 0.001

* Quartile 1 was considered as the reference.

Table 5 presents the results of logistic regression for anemia. Among the lipid‐based obesity indices, higher AIP was significantly associated with lower odds of anemia (Q4: 0.65; 95% CI: 0.49–0.88; P for trend = 0.01). Although Q4 of VAI was associated with lower odds of anemia in both males and females (males: 0.74; 95% CI: 0.55–0.99; females: 0.73; 95% CI: 0.55–0.99), the P for trend was not statistically significant. In contrast, higher LAP in both genders was associated with higher odds of anemia, and both the confidence intervals and P for trend were statistically significant. Among anthropometric parameters, HC, BAI, and ABSI were associated with 1.34‐fold (95% CI: 1.09–1.70; P for trend = 0.01), 1.39‐fold (95% CI: 1.10–1.86; P for trend = 0.01), and 2.6‐fold (95% CI: 1.68–3.03; P for trend < 0.001) higher odds of anemia in Q4, respectively. BMI showed an inverse association with anemia odds (P for trend = 0.02). WC also showed a significant positive trend with anemia, although the quartile‐specific estimates were not statistically significant.

**Table 5:** Multivariable logistic regression analysis of anemia based on lipid‐based obesity parameters and anthropometric indices quartiles.

*Variables Quartiles*	Anemia (OR & 95% CI)					
	Lap‐M	Lap‐F	VAI‐M	VAI‐F	CMI	AIP
Q2*	1.28 (0.97_1.61)	1.22 (0.93_1.61)	0.82 (0.64_1.04)	0.80 (0.63_1.03)	0.88 (0.67_1.17)	0.70 (0.55_0.90)
Q3	1.63 (1.23_2.14)	1.61 (1.22_2.11)	0.80 (0.62_1.03)	0.79 (0.62_1.09)	1.51 (1.16_1.97)	0.77 (0.60_0.99)
Q4	1.60 (1.20_2.13)	1.55 (1.16_2.06)	0.74 (0.55_0.99)	0.73 (0.55_0.99)	1.17 (0.89_1.54)	0.65 (0.49_0.88)
P for Trend	0.003	0.005	0.05	0.05	0.02	0.01
	BMI	WC	HC	ABSI	WWI	BAI
Q2	0.89 (0.66_1.20)	0.94 (0.73_1.22)	1.07 (0.82_1.39)	1.65 (1.22_2.22)	1.09 (0.84_1.42)	1.15 (0.88_1.50)
Q3	0.89 (0.65_1.16)	1.12 (0.87_1.43)	1.15 (0.89_1.48)	1.92 (1.43_2.57)	1.27 (0.97_1.65)	1.25 (0.95_1.63)
Q4	0.71 (0.51_0.93)	1.25 (0.97_1.57)	1.34 (1.09_1.70)	2.26 (1.68_3.03)	1.23 (0.93_1.62)	1.39 (1.10_1.86)
P for Trend	0.02	0.03	0.01	< 0.001	0.08	0.01

* Quartile 1 was considered as the reference.

**Figure 1:**
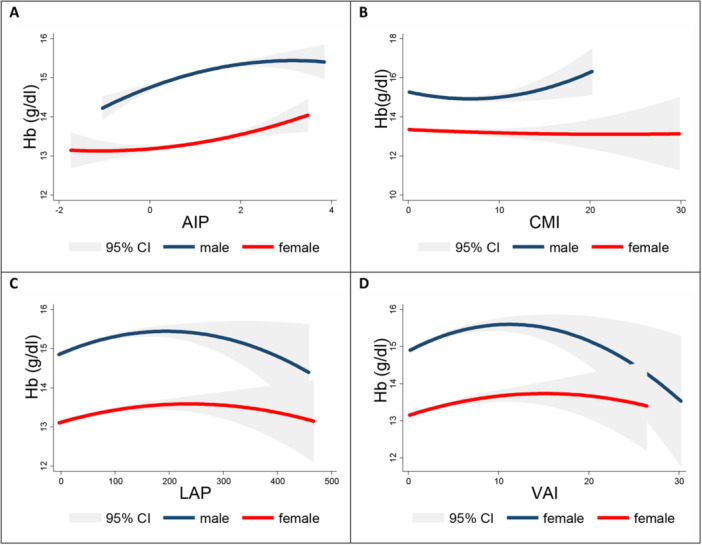
Quadratic fit plots with 95% confidence interval (CI) showing the association between lipid‑based obesity indices and Hb concentration, stratified by gender. (a) Atherogenic Index of Plasma (AIP) in both males and females with Hb concentration. (b) Cardiometabolic Index (CMI) in both males and females with Hb concentration. (c) Lipid Accumulation Product (LAP) in both males and females with Hb concentration. (d) Visceral Adiposity Index (VAI) in both males and females with Hb concentration.

**Figure 2:**
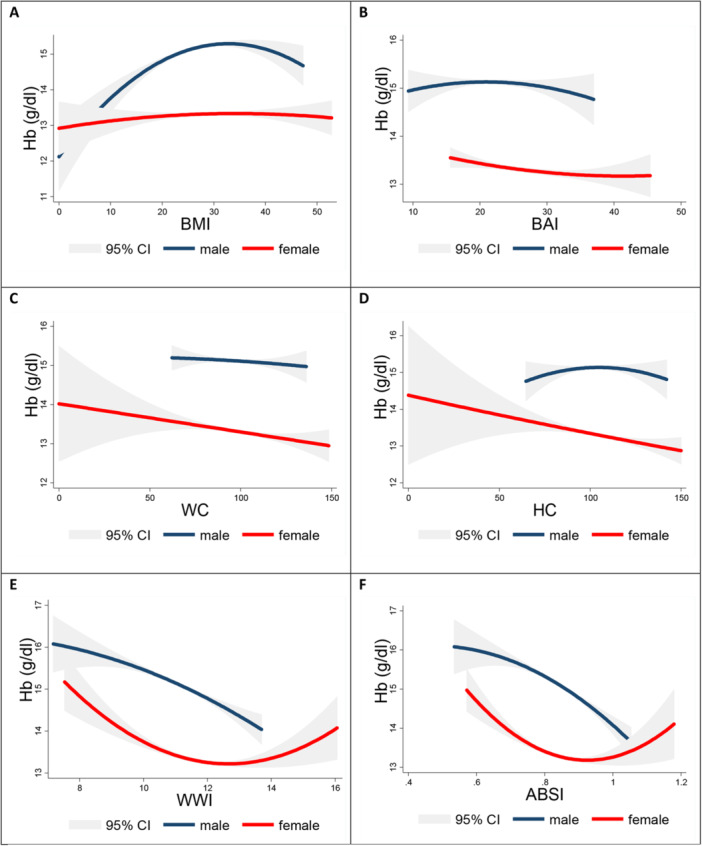
Quadratic fit plots with 95% confidence interval (CI) showing the association between anthropometric indices and Hb concentration, stratified by gender. (a) Body Mass Index (BMI) in males with BMI < 30 with Hb concentration. (b) Body Adiposity Index (BAI) in both males and females with Hb concentration. (c) Waist circumference (WC) in both males and females with Hb concentration. (d) Hip circumference (HC) in both males and females with Hb concentration. (e) Weight‑Adjusted‑Waist Index (WWI) in both males and females with Hb concentration. (f) A Body Shape Index (ABSI) in both males and females with Hb concentration.

Figures 1 and 2 show the quadratic fit plots by gender. AIP in both genders and CMI in males were positively associated with Hb concentration. In contrast, WC and HC in females, as well as WWI and ABSI in both genders, were negatively associated with Hb level. However, with the increasing WWI and ABSI, this negative association became stronger. In males with BMI < 30, Hb concentration increased with BMI, whereas this pattern was not observed in females or in males with BMI ≥ 30.

## 8 Discussion

The present study has investigated the association between anthropometric and lipid‐based indices with anemia in the Kurdish population of Iran. The results revealed a link between lipid accumulation and local obesity. Increasing WC showed a positive trend with higher odds for anemia; however, we found no significant association in each quartile. Also, a similar trend was found for HC and BAI, considering that the result in Q4 was statistically significant, along with P for the trend. The ABSI had a most considerable and significant association with anemia odds in our population. In contrast, parameters representing overall obesity, such as BMI and AIP, showed a protective effect against anemia; VAI in both genders also illustrated a protective influence, but the analysis for the trend was not statistically significant. Notably, we found LAP as an exception, which was a visceral obesity factor and significantly raised the odds of anemia.

The correlation between obesity and anemia remains inadequately comprehended. Obesity is associated with persistent low‐grade systemic inflammation. Obesity‐induced inflammation results in diminished oxygen levels and subsequently promotes the progressive development of inflammatory mediators, including C‐reactive protein (CRP), interleukin‐6 (IL‐6), interleukin‐10 (IL‐10), and interleukin‐1 beta receptor (IL‐1BR) [8].

Inflammation has a major role in anemia pathogenesis. Inflammation deprives iron storage, subsequently decreasing the average life span of erythrocytes [7]. We did not measure the specific inflammatory markers; nevertheless, examination of RDW‐CV as an inflammation indicator [23] showed that the anemic individuals had significantly higher RDW‐CV levels than non‐anemic individuals. Studies on the relationship between obesity and anemia emphasize the role of obesity in elevating the hepcidin concentration. Hepcidin is produced by adipose tissue in addition to hepatocytes [24]. The synthesis of hepcidin is upregulated in adipocytes of obese individuals by leptin [25, 26]. The plasma release of ferrous iron from duodenal enterocytes, iron recycling macrophages, and hepatocytes is reduced during inflammatory conditions as a result of the increase in hepcidin through endocytosis and proteolysis of ferroportin. Consequently, the iron stores as ferritin in the cytoplasm of these cells are reduced [27]. However, the findings of research on hepcidin are also contradictory. Siddique et al. studied 675 adults over the age of 18 who had non‐alcoholic fatty liver disease and obesity. They discovered that serum hepcidin levels were substantially lower in individuals with iron deficiency [10]. Furthermore, Rodríguez‐Mortera and colleagues asserted that the elevation of hepcidin was more closely associated with metabolic and inflammatory changes than with iron homeostasis [28].

Given the established associations between obesity, insulin resistance, and cardiovascular disease [29], this study investigates the association between obesity and anemia, focusing on different types of obesity: local, general, and visceral. Very few studies have been conducted on this subject. Komolova et al., by examining rats, reported that prenatal iron deficiency is associated with visceral adiposity and might be a potential risk factor for cardiometabolic dysfunction in offspring. In 2020, Tanaka et al. reported that reduced visceral fat was associated with lower mean corpuscular volume (MCV) in non‐anemic individuals [30]. The current study demonstrated that the mean VAI was significantly lower in the anemic group compared to the non‐anemic group. Higher VAI levels were positively associated with RBC count, Hb, and HCT but inversely associated with MCH, MCHC, and MCV. Additionally, elevated VAI was associated with increased RDW‐CV in both genders. However, logistic regression analysis revealed that higher VAI was associated with a reduced odds of anemia, especially in Q4. Nonetheless, the LAP, as an indicator of visceral adipose tissue [31], was found to have a link with higher anemia in both males and females. Several investigations indicated that visceral adiposity exhibits a weaker association with anemia compared to peripheral obesity [32]. The observed contradictory result may be attributed to differences in inflammatory profiles. In our population‐based study, analysis of the DII across LAP quartiles showed that individuals in the fourth LAP quartile (both males and females) had significantly higher DII scores (see Supplementary Table 1). Additionally, a positive association was observed between LAP and RDW‐CV, though the effect size was small. Further investigation of specific inflammatory parameters is needed to elucidate this relationship.

The literature mainly focuses on BMI concerning anemia risk. An investigation on Mexican women showed that obesity could increase anemia risk 2–4 times [33]. Similarly, the study on the Iranian population reported that higher BMI was associated with an increased anemia prevalence [12]. In contrast, Ausk et al. found no significant impact of BMI on hemoglobin levels or anemia incidence between normal‐weight and obese individuals [7]. Additionally, a Chinese study by Qin et al. identified an inverse relationship between BMI, central obesity, and anemia risk [4]. Our study observed no BMI differences across groups, aligning with Qin et al.'s findings after confounder adjustment. Logistic regression analysis indicated that higher BMI quartiles were associated with a reduced anemia prevalence. Moreover, the relationship between BMI and red blood cell indices had the same pattern observed with VAI.

Conversely, anthropometric indices reflecting localized fat accumulation, such as WC, HC, BAI, and ABSI, were elevated in the anemic group compared to the non‐anemic group. Linear regression analysis indicated a positive trend between these indices and RDW‐CV, alongside a negative trend with RBC count, HCT, and HGB. These indices were associated with an increase in odds of anemia, with statistically significant results observed for ABSI, BAI, and HC (*P* for trend < 0.05). Research has established a link between adipose tissue accumulation and inflammation [34]. Individuals with abdominal obesity exhibit a heightened proatherogenic profile, potentially elevating inflammatory markers and adipokines, including tumor necrosis factor‐alpha (TNF‐α) and an increased adiponectin‐to‐leptin ratio [35].

Moreover, the quadratic fit plot employed in this study indicated that increases in WC and HC had a more negative effect on HGB concentration in females. Conversely, elevations in the WWI and ABSI were negatively correlated with HGB levels in the male population. Growing evidence indicates that women typically possess 10% more body fat than men, with a greater proportion stored as subcutaneous adipose tissue (SCAT) in the abdominal and gluteofemoral regions [36]. Research suggests this disparity is influenced by sex‐specific variations in genetic, epigenetic, hormonal, and inflammatory factors, although the precise mechanistic pathways remain incompletely defined [37]. Furthermore, inconsistent findings in anemia research may be explained by the diversity of adiposity patterns and fat distribution across different populations [33].

Consequently, the increased prevalence of anemia observed in females may align with existing literature due to distinct body fat distribution. The current study's findings support this, showing that 72% of female participants had anemia, compared to 27% of males. In the context of anemia, adiposity patterning may therefore serve as a more relevant predictive factor than total body mass for estimating anemia incidence.

This study is the first to evaluate the anthropometric indices WWI, ABSI, and BAI in an Iranian ethnic group with anemia. It also represents the first assessment of the lipid‐based obesity parameters AIP, CMI, LAP, and VAI in a relatively large study population from this group.

However, several limitations must be acknowledged. Firstly, the cross‐sectional design of this study must be confirmed by the results of longitudinal research. Secondly, the analysis was restricted to common CBC parameters and lacked more specific blood and inflammatory markers. A more comprehensive assessment, including measures of transferrin, ferritin, and tissue iron stores alongside CRP, IL‐6, etc., is required to elucidate the precise influence of these indices on anemia risk.

## 9 Conclusion

The present study demonstrated that parameters associated with localized obesity, including WC, HC, WWI, BAI, and ABSI, are more associated with anemia in the Iranian Kurdish population. Notably, ABSI demonstrated the strongest association, with a 2.26‐fold increase in the odds of anemia. Conversely, BMI, along with visceral obesity markers such as VAI and AIP, was inversely related to the anemia. An exception was observed for LAP in both males and females, where participants in the fourth quartile showed a significantly higher chance for anemia compared to those in the first quartile. This finding may be attributed to differences in inflammatory profiles. These results suggest that body shape and localized fat distribution may have a greater association with anemia development than overall obesity. Therefore, anthropometric indices reflecting localized obesity could serve as more effective and practical tools for assessing anemia risk.

## Author Contributions

**Maryam Kohsari:** writing – original draft, formal analysis, investigation, data curation. **Zohreh Rahimi:** writing – review and editing, validation. **Mehdi Moradinazar:** conceptualization, methodology, formal analysis, supervision, writing – review and editing, investigation, validation.

## Conflicts of Interest

The authors declare no conflicts of interest.

## Author Declaration

All authors have read and approved the final version of the manuscript. Mehdi Moradinazar had full access to all of the data in this study and takes complete responsibility for the integrity of the data and the accuracy of the data analysis.

## Transparency Statement

The corresponding author, Mehdi Moradinazar, affirms that this manuscript is an honest, accurate, and transparent account of the study being reported; that no important aspects of the study have been omitted; and that any discrepancies from the study as planned (and, if relevant, registered) have been explained.

## Supporting information


Supporting File

## Data Availability

The data that support the findings of this study are available on request from the corresponding author. The data are not publicly available due to privacy or ethical restrictions.
