# Supplementary material for: Investigating the Association Between Anemia and Biochemical Factors in Relation to New Anthropometric Metrics in an Iranian Cohort: A Cross‐Sectional Study
Source: Health Sci Rep. 2026 Jul 30;9(8):e72867. doi: 10.1002/hsr2.72867 (PMC13421792; doi:10.1002/hsr2.72867)
Supplement: Supplementary file 1 — Supporting File [file HSR2-9-e72867-s001.docx]

Table S1. The mean of DII based on LAP quartiles.

| **Variable** | **LAP-Male** | | | | **P-value** | **LAP-Female** | | | | **P-value** |
| --- | --- | --- | --- | --- | --- | --- | --- | --- | --- | --- |
| **DII** | **Q1** | **Q2** | **Q3** | **Q4** |  | **Q1** | **Q2** | **Q3** | **Q4** |  |
|  | -1.64±1.72 | -2.37±1.53 | -2.54±1.50 | -2.73±1.38 | <0.001 | -1.71±1.71 | -2.35±1.55 | -2.53±1.51 | -2.70±1.39 | <0.001 |
